# Stunting, IQ, and final school attainment in the Cebu Longitudinal Health and Nutrition Survey birth cohort

**DOI:** 10.1016/j.ehb.2021.100999

**Published:** 2021-08

**Authors:** Linda S. Adair, Delia B. Carba, Nanette R. Lee, Judith B. Borja

**Affiliations:** a Department of Nutrition, Gillings School of Global Public Health, Carolina Population Center, CB #8120, University of North Carolina at Chapel Hill, Chapel Hill, NC, 27599, United States; b USC-Office of Population Studies Foundation, Inc., University of San Carlos, Cebu City, Philippines

**Keywords:** School attainment, Schooling continuation, Stunting, IQ, Philippines, Cohort study

## Abstract

School attainment is an important aspect of human capital, and a key determinant of long-term health and well-being. Early life deprivation and poor nutritional status are well known predictors of school entry and progression. We examine the persistence of early life influences and subsequent socioeconomic disadvantage (SED) across the multiple school continuation decisions that lead to final school attainment. Using data from a Philippine birth cohort followed for 35 years, we model 6 continuation decisions: Did not complete elementary school, elementary graduate only (completed grade 6), some secondary schooling, high school graduate, some postsecondary schooling, and college graduate, as well as total years of schooling. We estimate the association of school attainment with early life length for age Z-score (LAZ at 2 years of age) and cognitive development (IQ) as well as underlying indicators of SED and other family influences through early adulthood. The analysis sample includes >1900 participants in the Cebu Longitudinal Health and Nutrition Survey. Females completed, on average, one year more schooling than males, and twice as many females as males were college graduates (29.1 vs 15.0 %). LAZ and one standard deviation of IQ were each independently associated with 0.4 more years of attained schooling. A path model demonstrated strong direct associations of SED with years of schooling as well as indirect associations through LAZ and IQ. Sequential logits used to estimate continuing education decisions show persistent associations of early life LAZ and IQ and schooling even after accounting for changing SED of households over the schooling life course. Filipino parents had high but often unmet educational aspirations for their children because of the child’s loss of interest in school and perceived financial barriers. Results further emphasize the importance of early life SED as a key risk factor for suboptimal school attainment.

## Highlights

• CLHNS females were more likely than males to stay in school across all major thresholds from completing elementary school to college graduation.
• Stunting at age 2 and IQ score at age 8 are independently related to final years of schooling (0.4 years per SD of LAZ and IQ).
• Early life LAZ and IQ remain strong predictors of school attainment after accounting for changing household disadvantage as children get older.
• Preschool/kindergarten attendance is associated with more attained schooling in both stunted and non-stunted children.

## 1 Introduction

School attainment is a key component of human capital, and influences a broad spectrum of health, social, and economic outcomes. The importance of school attainment is underscored in the United Nation’s 2030 Agenda for Sustainable Development, where Goal 4 includes targets to ensure that all children have access to quality early childhood development opportunities and preprimary education and free, equitable and quality primary and secondary education (United Nations, 2017). An affordable, high quality higher education is also envisioned. In today’s world, post-secondary education is particularly important to foster the development agendas of low- and middle-income countries (LMIC). Higher education contributes to a greater likelihood of being in the labor force, higher individual incomes and more social capital thereby contributing to national development (Shafiq et al., 2019), and potentially escaping poverty. Thus, it is important to identify the factors that contribute to continuation of schooling from enrollment to higher education degrees.

Multiple aspects of poor school attainment, including delayed school entry, grade repetition, and decreased final grade attainment are related to poor child health and nutritional status early in life. A recent focus has been on the first 1000 days, encompassing the time from conception through the first 2 years of life when rapid growth and development are occurring. Evidence comes largely from studies that relate school attainment to early life stature, a measure of chronic deprivation. For example, a pooled analysis of data from five LMIC revealed a 0.5 year deficit in final years of schooling for each 1 unit lower length-for-age Z-score (LAZ) at 2 years of age (Victora et al., 2008). In the Young Lives study, Crookston and colleagues (Crookston et al., 2011) reported that persistent stunting doubled the likelihood of being older than expected given grade in school, most often because of delayed school entry. In our own prior work in the Philippines, we showed that lower LAZ at 2 years of age was associated with older age at school enrolment, increased likelihood of grade repetition, and decreased likelihood of completing high school by age 18 (Daniels and Adair, 2004). These associations remain, even after controlling for parental education and other socioeconomic predictors of stunting. In general, stunting is not regarded as causally related to school attainment. The economics literature notes the potential for biased estimates of this relationship when caregivers adjust their investments in child health in response to child characteristics such as height (Liu et al., 2009), (Glewwe, 2005; Glewwe et al., 2001) (Alderman et al., 2001, 1999) (Behrman et al., 2017).

At the same time, many measured and often unmeasured underlying aspects of chronic deprivation that limit physical growth also impair cognitive development, (Leroy and Frongillo, 2019; Perkins et al., 2017), resulting in a strong association of early child stunting with IQ or other measures of cognitive capacity (Prado et al., 2019a, b; Chang et al., 2002) (Walker et al., 2015; Casale et al., 2014). Child cognitive capacity is also related to school attainment. Based on a large meta-analysis, Prado and colleagues identify unique and shared underlying risk factors for physical versus cognitive development (Prado et al., 2019a), with important implications for the design and implementation of interventions to improve both outcomes.

Even if the associations of early life stunting, cognitive development and school attainment are not causal, understanding the pathways through which they are associated can be informative for generating hypotheses and identifying possible areas for intervention.

With a focus on early life, there is a missed opportunity to study inputs to the multiple schooling decisions made as children get older, and which together determine final school attainment. Such inputs may represent continued constraints to schooling or enablers of progression that may modify how early life factors relate to schooling outcomes. For example, illness or family social and economic factors such as parental unemployment, birth of younger siblings, or loss of a parent, may hinder school continuation even among well-nourished children. Conversely, continued parental investments may encourage school continuation even among poorly nourished children. Child cognitive abilities and prior schooling success may enhance the likelihood of school continuation irrespective of nutritional status. A better understanding of early life and later age-specific inputs may inform more effective policies and interventions tailored to promote school continuation at each stage of schooling.

The main aim of this paper is to identify key factors related to school continuation at each critical education landmark, including completion of elementary school, completion of secondary school, progression to post-secondary schooling, including vocational training, and completion of a higher education degree. We estimate the association of school continuation with early life stunting and IQ, aiming to understand the persistence of associations of early life factors across the range of school continuation decisions. We then explore how time-varying inputs in early- to mid-childhood, adolescence, and young adulthood relate to continuation at each stage and test whether such inputs mitigate or exacerbate early life deficits. For comparison with other studies, we estimate a model of final years of schooling, and use a path model to provide insight on the interrelationships among early life disadvantage, impaired physical growth, poor cognitive development and school attainment.

## 2 Methods

### 2.1 Sample and data

Data come from the Cebu (Philippines) Longitudinal Health and Nutrition Survey (CLHNS) (Adair et al., 2011). The CLHNS is a community-based study that recruited 3327 women from 33 randomly selected *barangays* (administrative units) from the 252 barangays that comprised Metro Cebu in 1982. Singletons born in a one-year (1983−84) period (n = 3080) form the cohort of index children followed to age 35 years, with surveys every 2 months for the first 2 years, and follow-up surveys in 1991, 1994, 1998, 2002, 2005, 2009 and 2018. All surveys and data protections were carried out in accordance with The Code of Ethics of the Declaration of Helsinki, with IRB approvals obtained from the University of North Carolina at Chapel Hill, and the University of San Carlos Research Ethics Committee, Cebu, Philippines.

To characterize early life experiences, we use data from the baseline visit in the 6th to 7th month of pregnancy, a survey within several days of birth, and bimonthly postnatal visits. Follow-up surveys were at approximate ages of 8, 11, 15–16, 18, 21, 26, and 35 years.

Beginning with the 1991 survey (mean age 8.5 yr), schooling data collected at each follow-up visit included current enrollment status, schooling history, type of school(s) attended, and reasons for discontinuation. With the 2018 data, we assume that school attainment is final since few participants are expected to return to school after age 35 years. Prior publications using the CLHNS data included participants who were still in school. The Philippine educational system during the study years of our sample was structured for 6 years of elementary education and 4 years of high school with usual age of 6–7 years at entry into grade 1. We define two types of schooling outcomes: (1) Years of schooling as a continuous variable ignoring grade repetition, where 10 years indicates completion of high school, and (2) Six attainment categories: did not complete elementary school, elementary graduate only (completed grade 6), some secondary schooling, high school graduate, some postsecondary schooling, and college graduate or higher). Participants lost to follow-up have unknown final school attainment. The analysis excludes those lost to follow-up who were still enrolled in school at their last survey visit. However, we included individuals who had dropped out prior to their last survey visit and assumed that their last grade attended represented their final grade.

Child and family characteristics assessed at birth include child’s birth order, birth weight and length, and gestational age (estimated from the woman’s last menstrual period date and/or Ballard clinical assessment of newborns). Length-for-age Z score close to 2 years of age or a binary variable representing stunting (LAZ<-2, calculated from the WHO growth standards) represent cumulative influences on child nutrition during the first 1000 days. We used the 24-month Z-scores for most participants and substituted the mean of Z-scores at 20 and 22 months when the 24 -month value was missing (n = 56), since Z-scores changed little during that time period. Early life socioeconomic status was represented by mother’s and father’s education, and the average of measures obtained at baseline and when the child was near 2 years of age for household size, total household income, household assets (a score from 0 to 11 representing house ownership and construction materials, and ownership of appliances and vehicles), and presence of “readers” (individuals who read books, magazines, or newspapers) in the home. Father’s schooling is missing for most fathers not living in the household at the time of the survey. We added a separate category of missing father to the father’s schooling variable to account for this pattern of missingness. We include community level indicators of urbanicity derived from community surveys among key informants concerning schools, markets, transportation, communications, health infrastructure, population size and population density (Dahly and Adair, 2007).

Child IQ was measured at a mean age of 8.5 years using a test of fluid ability developed and validated for use in Filipino children (Guthrie et al., 1977). The test comprises 100 cards, each with drawings of 5 objects. The child is asked to select which of the 5 is different from the other 4. The score reflects the number of correct answers. The IQ test was administered when most study participants were already in school, and the majority were in grade two. IQ scores were significantly correlated with time in school (0.27, p < 0.001). Since we need a measure of IQ that is independent of schooling, we regressed IQ on school grade at the time the test was administered, and use the residual of IQ, internally standardized to the full sample with complete test results (residual IQ Z-score, rIQ).

Major life events for the child or family after age 2 include moving, separation of mother and child (owing to death or other reasons), changes in wealth and income, birth of younger siblings, presence of older siblings, major injuries or illnesses requiring hospitalization, and for adolescents and young adults, pregnancy and marriage. To add context to the quantitative analysis, we also describe parenting behaviors that support child learning, including reading to the child and helping the child with homework; schooling aspirations; and parent and child reasons for discontinuing schooling.

### 2.2 Statistical analysis

Since final schooling represents multiple continuation decisions across the child’s life, each building on the prior decision (Mare, 1981), we use sequential logit models to estimate the likelihood of continuing to the next level of schooling among those who passed the prior level. This allows us to determine whether early life factors relate differently to each of the six continuation outcomes. The analysis, which is comprised of 6 logistic regression models, was implemented in Stata 16 (StataCorp, 2016) with the SEQLOGIT add-on for convenience but could simply be estimated using 6 separate logistic regressions (Buis, 2007).

We then consider the role of ***time varying*** constraints or enablers encountered over time ***in addition to*** the early life factors. Since SEQLOGIT does not accommodate time-varying covariates, we separately estimated 6 logistic regression models to determine the likelihood of continuing to each next level of schooling, given completion of the prior, accounting for early life LAZ, birth order, parental education, mother’s height, and child sex as above, but we substitute contemporary for early life socioeconomic circumstances, and add health, and other life events measured at the survey preceding the school continuation decision.

To assess the role of rIQ, we estimate each of the models described above, alternately including rIQ, separately and then with LAZ.

In 1991, 2264 index children remained from the baseline sample of 3080 singleton live births. Loss to follow-up reflects deaths (n = 209), migration out of the Metro Cebu area (n = 580), refusals (n = 13), and other reasons (n = 14). From this sample, we excluded children who were subsequently lost to follow-up if they were still attending school at the last survey for which we have data, and those who were missing an early life length measure, leaving an analysis sample of 1,958. IQ was measured in 1934 of these children. We address the role of attrition and missing data using inverse probability weighting (Seaman and White, 2013). We first estimate a probit model to predict who is in the LAZ sample (n = 1958) or not (n = 326), specified with exogenous variables representing household SES, demographic, and environmental characteristics in early life. We use the inverse of the probability of inclusion as a sample weight (IPW) and compare weighted and unweighted models of final years of schooling to estimate the overall impact of missing data. This approach ignores attrition between the 1983−84 baseline survey and the 1991−94 follow-up surveys because our focus in on a school-age sample.

For comparison with most of the literature on stunting and schooling, we estimate how severity of child stunting at 2 years of age and rIQ at 8 years of age relates to the total completed years of schooling (ignoring grade repetition) using linear regression. We include early life household and family characteristics (maternal and paternal education; household size, income; and assets; presence of readers in the home; child sex and firstborn status) and community urbanicity as potential confounders.

Finally, we estimate a path model to show direct and indirect pathways through which socioeconomic disadvantage (SED) relates to final schooling. In this model, we treat SED as a latent variable measured by parental education, household size, income, assets, urbanicity, firstborn status, maternal height and readers in the home and specify paths from the latent SED variable to final schooling through IQ and LAZ, allowing for a direct path from LAZ to schooling as well as an indirect path through IQ. The model adjusts for sex of the child and whether the child had started school when the IQ test was administered.

## 3 Results

### 3.1 Sample characteristics

Differences between the analysis sample (n = 1958) and those excluded (n = 326), largely reflects the higher income, assets, and education of those who left the Metro Cebu area before completing their schooling (Table 1). Despite these differences, comparison of estimates with and without inclusion of IPWs were mostly different only at the second decimal place. We therefore ran final models without IPWs.

**Table 1:** Characteristics of CLHNS participants Excluded or Included from the analytic sample.

Variable	N (excluded)	Mean or proportion	SD	N (Included)	Mean or proportion	SD	p-value
Years schooling	326	6.94	4.47	1958	9.97	3.13	<0.001
LAZ	231	−2.31	1.12	1958	−2.56	1.11	0.001
IQZ	318	53.89	12.75	1934	51.01	12.42	<0.001
Stunted	231	0.61		1958	0.68	0.47	0.030
Firstborn	326	0.25		1958	0.22	0.41	0.222
Birth length (cm)	324	49.28	1.89	1957	49.11	2.03	0.159
Birth weight (kg)	322	3.00	0.41	1928	3.01	0.42	0.879
Gest. Age (weeks)	326	38.54	1.92	1941	38.40	2.02	0.265
Urbanicity score	326	32.94	12.30	1958	28.53	12.96	<0.001
Assets Score	326	3.30	2.17	1958	2.51	1.81	<0.001
HH Income (pesos/week)	326	312.98	310.55	1958	236.68	216.29	0.032
HH size	326	5.87	2.97	1958	5.74	2.79	0.475
Maternal age (yr)	326	26.19	6.17	1958	26.06	6.03	0.713
Maternal height (cm)	326	151.20	5.51	1958	150.49	4.93	0.019
Mother's schooling							<0.001
< 6 years	326	0.21		1958	0.32		
Elementary only (6 years)		0.21			0.26		
Some High School		0.33			0.29		
High School Grad		0.25			0.13		
Father's schooling							<0.001
Missing	326	0.09			0.05		
< 6 years		0.18		1958	0.30		
Elementary only (6 years)		0.15			0.22		
Some High School		0.32			0.29		
High School grad		0.26			0.14		
Readers in the home	326	0.71		1958	0.61		<0.001

**Fig. 1:**
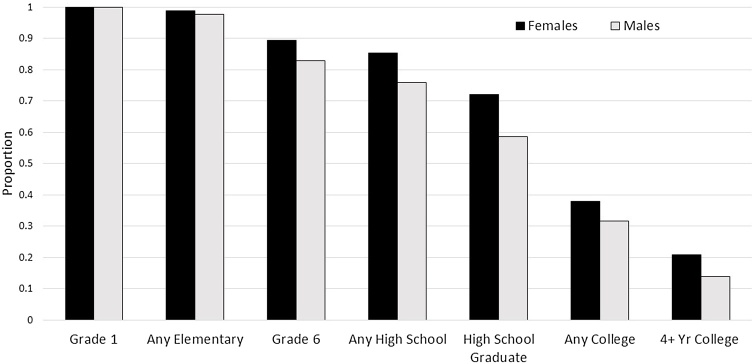
School continuation rates for males and females in the CLHNS, n = 1958.

In the analysis sample of 1958, all but 9 completed at least one year of elementary school. Males completed, on average 9.47 (SD 3.34) years of schooling, while females completed 10.52 (SD 2.77) years. The gender gap widened for entering and completing high school, then narrowed among those who continued to attain college education (Fig. 1). In terms of final school attainment, 78.5 % of females and 62.1 % of males were high school graduates (including those who continued with additional schooling after high school), 32.6 % of males and 39.7 % of females had some postsecondary education, and 15.0 % of males and 29.1 % of females were college graduates. Among those present in 1994 when the question was first asked (n = 1935), 38.9 % of males and 43.1 % of females had ever attended preschool or kindergarten.

### 3.2 Likelihood of progression to the next level of schooling

Odds ratios from the sequential logits represent the likelihood of continuing to the next level of schooling *among those who completed the prior level*. Table 2 summarizes results from school continuation models with early life LAZ and rIQ. The first set of estimates account for early life factors only, while the second set includes time-varying factors. Full model results for the logits are presented in supplementary **tables 1 and 2**.

**Table 2:** Sequential logits^1^ relating early life LAZ and IQ to school transitions (N = 1934 with LAZ and IQZ).

	Early Life Only		With Time-Varying Covariates	
	Odds Ratio	95 % CI	Odds Ratio	95 % CI
**Completed elementary**				
LAZ	1.33***	1.13,1.57	1.22*	1.03,1.45
LAZ adjusted for IQ	1.35***	1.14,1.6	1.23*	1.03,1.48
IQZ	1.20	0.99,1.44	1.13	0.93, 1.37
IQ adjusted for LAZ	1.17	0.97,1.41	1.11	0.92, 1.35

**Some high school**				
LAZ	1.20	0.99,1.46	1.12	0.92,1.37
LAZ adjusted for IQ	1.15	0.94,1.40	1.07	0.87,1.31
IQZ	1.40**	1.13,1.73	1.34**	1.08,1.66
IQ adjusted for LAZ	1.38**	1.12,1.71	1.33**	1.08, 1.65

**High school graduate**				
LAZ	1.22**	1.07,1.39	1.22**	1.06,1.39
LAZ adjusted for IQ	1.21*	1.05,1.38	1.20**	1.04,1.38
IQZ	1.20*	1.04,1.39	1.16**	1.00,1.35
IQ adjusted for LAZ	1.19*	1.03,1.37	1.15	0.99,1.33

**Some college**				
LAZ	1.34***	1.18,1.52	1.40***	1.22,1.60
LAZ adjusted for IQ	1.30***	1.15,1.48	1.36***	1.19,1.56
IQZ	1.35***	1.19,1.54	1.31***	1.14,1.49
IQ adjusted for LAZ	1.33***	1.17,1.51	1.28***	1.11,1.46

**College graduate**				
LAZ	1.19	1.00,1.41	1.17	0.98,1.39
LAZ adjusted for IQ	1.17	0.98,1.40	1.16	0.97,1.38
IQZ	1.21*	1.02,1.42	1.21*	1.02,1.44
IQ adjusted for LAZ	1.20*	1.0,1.41	1.21*	1.01,1.44

1. All models included sex, firstborn status, mother’s and father’s education, household size, income and assets, urbanicity and presence of readers.

*p < .05, **p < 0.01 ***p < 0.001.

In the model considering only early life factors, mutual adjustment for LAZ and rIQ had little impact on estimates, that is, the association of LAZ with schooling outcomes was not substantially changed by the inclusion of rIQ. Adjusted for rIQ, each unit of LAZ was associated with a 35 % increase in the likelihood of completing elementary school, a nearly 20 % increase in likelihood of starting or completing high school and completing college and a 30 % increase in change of attending but not completing a college degree. While similar in magnitude, the precision of these estimates varies. rIQ was unrelated to completion of elementary school but positively associated with each subsequent transition, with coefficients quite similar to those for LAZ. Males were less likely than females to continue to each subsequent level, Firstborn status, urbanicity, and presence of readers were not significantly related to continuation to any level. Early life household income was associated with the completion of elementary school and some high school, while household assets significantly predicted only continuing from high school to college. Parental education associations with school continuation were all positive, but differed in magnitude and significance across transitions, and variation in whether mother’s or father’s mattered more.

When time varying factors were included, the associations of school continuation with LAZ and rIQ were relatively unchanged, but time-varying household assets were significant and stable predictors of continuation at all levels. Household income, hospitalizations, and number of older siblings in the household were unrelated to any transitions. Addition of younger siblings reduced the likelihood that elementary school graduates would go on to high school; presence of the mother (vs absence) in the household was important for high school graduation and moving reduced the likelihood of finishing elementary school. Marriage markedly reduced the likelihood that a high school graduate would go on to college among males and females.

### 3.3 Preschool/kindergarten

We ran separate models which added having attended a preschool or kindergarten to the early life model reported above, and found that attendance was significantly associated with increased likelihood of completing elementary school (OR = 1.50, 95 % CI 1.18,1/89), of completing some high school (OR = 1.43 95 % CI 0.99,2.08) and of continuing past high school (OR = 1.47, 95 % CI 1.13, 1.92). Inclusion of having preschool or kindergarten in the model had little effect on other coefficients in the model.

### 3.4 Total school years

Completed years of schooling are strongly associated with early life LAZ and rIQ (Table 3). Each SD increase in LAZ is associated with 0.40 years additional schooling, while 1 SD of rIQ is associated with 0.39 years. From these coefficients we can estimate that nearly a full year of total schooling separates the most severely stunted children (LAZ<-3) from those with LAZ>-1, after adjustment for early life circumstances. Inclusion of rIQ with LAZ in the regression model has little effect on the association of LAZ with years of schooling (from 0.40 to 0.37 years). Other strong positive predictors of years of schooling include higher household assets and income, higher levels of maternal and paternal schooling, and smaller household size. While there are marked male-female differences in years of schooling, non-signifcant interaction terms indicate that the relationship of early life LAZ and rIQ to years of schooling is not different in males compared to females.

**Table 3:** Linear regression of final years schooling on early life LAZ and IQ.

	LAZ		IQZ		LAZ + IQZ	
	b	95 % CI	b	95 % CI	b	95 % CI
LAZ	0.40***	0.28,0.52			0.37***	0.25,0.49
IQZ			0.39***	0.26,0.52	0.36***	0.23,0.481
Male	−1.13***	−1.37,-0.89	−1.17***	−1.41,-0.93	−1.12***	−1.36,-0.89
Firstborn	−0.01	−0.32,0.29	0.09	−0.22,0.39	−0.02	−0.32,0.28
Urbanicity score	0.00	−0.01,0.01	0	−0.10,0.10	−0.01	−0.11,0.10
HH Assets	0.20***	0.12,0.29	0.23***	0.14,0.31	0.20***	0.11,0.28
HH income Q1 (ref)						
HH Income Q2	0.18	−0.19,0.56	0.17	−0.21,0.54	0.20	−0.17,0.57
HH Income Q3	0.57**	0.19,0.95	0.53**	0.15,0.92	0.54**	0.17,0.92
HH Income Q4	0.77***	0.36,1.17	0.78***	0.37,1.18	0.76***	0.36,1.16
HH Income Q5	0.52*	0.05,0.99	0.53*	0.06,1.00	0.50*	0.03,0.96
HH size (# persons)	−0.07**	−0.11,-0.02	−0.08***	−0.13,-0.04	−0.06*	−0.11,-0.01
Maternal height (cm)	0.00	−0.03,0.02	0.02	−0.01,0.04	0.00	−0.03,0.02
Mom <6 years (ref)						
Elementary Only (6 years)	0.66***	0.33,0.99	0.59***	0.26,0.92	0.59***	0.27,0.91
Some HS	1.05***	0.69,1.40	0.98***	0.62,1.33	0.96***	0.61,1.30
HS graduate	1.90***	1.39,2.41	1.90***	1.40,2.41	1.77***	1.27,2.27
Dad missing educ	0.34	−0.27,0.96	0.35	−0.26,0.97	0.37	−0.24,0.99
Dad <6 years (ref)						
Elementary Only (6 years)	0.41*	0.06,0.75	0.35*	0.00,0.69	0.37*	0.03,0.71
Some HS	1.01***	0.66,1.36	0.97***	0.61,1.32	0.92***	0.57,1.27
HS graduate	1.50***	1.01,1.98	1.47***	0.98,1.96	1.41***	0.92,1.89
Readers in the home	0.20	−0.07,0.48	0.24	−0.03,0.51	0.21	−0.06,0.49

Intercept	10.25***	6.30,14.20	6.79***	3.25,10.33	9.74***	5.99,13.49
N	1958		1934		1934	

### 3.5 The role of improvement in linear growth between ages 2 and 8 years

Mean LAZ scores at 2 and 8 years of age were -2.56 and -2.08, respectively. We estimated an additional set of models that included change in length/height Z score (L/HAZ) from age 2 to age 8 years, along with early life LAZ, rIQ and the full set of early life covariates described above (Table 4). LAZ, change in L/HAZ, and rIQ were each independently associated with years of schooling. In the sequential logit models, LAZ and change in L/HAZ were both associated with higher likelihood of completing elementary school, but while early life LAZ remained as a significant predictor for all transitions except college graduation, change in L/HAZ was unrelated to later continuation.

**Table 4:** Improved linear growth^1^ and school attainment among CLHNS participants (n = 1934). Results from (A) linear regression models of total years of schooling and B) sequential Logit models that estimate the odds of continuing to the next school level, given completion of the prior level^2^.

A. Total years of schooling		
	b	95 % CI
Early life LAZ	0.51***	0.36, 0.66
LAZ change	0.31***	0.12, 0.49
ZIQ	0.35***	0.23, 0.47

B. Continuation		
	Odds Ratio	95 % CI
**Completed Elementary**		
Early life LAZ	1.68***	1.36,2.09
LAZ change	1.57**	1.20,2.07
ZIQ	1.16	0.97,1.40

**Some High School**		
Early life LAZ	1.33*	1.02,1.72
LAZ change	1.32	0.95,1.82
ZIQ	1.38**	1.12,1.71

**High School Graduate**		
Early life LAZ	1.31**	1.10,1.56
LAZ change	1.19	0.96,1.47
ZIQ	1.18*	1.03,1.37

**Some College**		
Early life LAZ	1.31***	1.12,1.53
LAZ change	1.01	0.83,1.23
ZIQ	1.33***	1.17,1.51

**College Graduate**		
Early life LAZ	1.13	0.92,1.39
LAZ change	0.93	0.71,1.20
ZIQ	1.20*	1.02,1.42

*p < .05, **p < 0.01 ***p < 0.001.

1. Change in Z-score (HAZ at age 8 years-LAZ at age 2 years).

2. Adjusted for sex, firstborn, urbanicity, household income, assets and size maternal height, parental education, readers.

### 3.6 Schooling, LAZ and IQ pathways in a path model (Fig. 2)

In this model, the latent SED variable has significant factor loadings on all of the indicators included in the measurement portion of the model. SED relates to final years of schooling directly and indirectly through LAZ and IQ. We also specify direct paths from maternal height to LAZ, and from sex to IQ, LAZ, final schooling and time in school at age 8. The latter is included because the IQ test was administered at age 8 after many children had started school, and a longer time in school is associated with better test performance. With the exception of the path from sex to time in school at age 8, all beta coefficients are significant at p < 0.004. SED paths to LAZ and IQ have similar coefficients (0.35, 0.36), and the direct association of SED with attained schooling is large (1.27). The IQ association with schooling (0.78) is nearly 4 times greater than that of LAZ (0.20). The model indicates a significant path from LAZ to IQ and thus an indirect association of LAZ to schooling. The total effect of LAZ on schooling in this model is 0.29 years schooling per LAZ. Male sex relates directly to years of schooling, and indirectly via lower LAZ, lower IQ, and less time in school at age 8, for a total effect of -1.2 years.

**Fig. 2:**
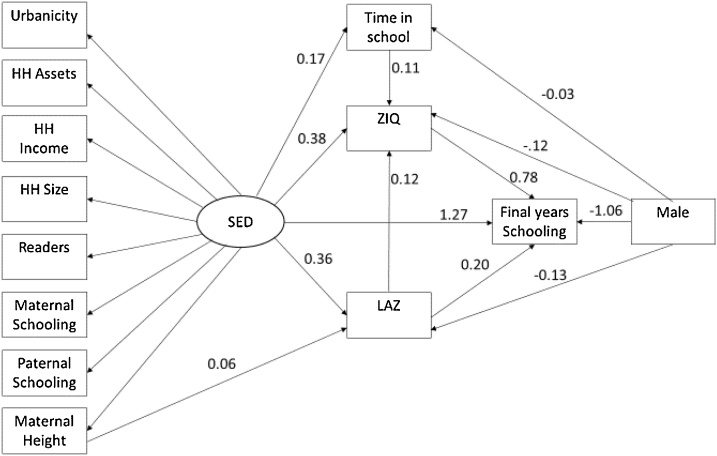
Path models relating early life socioeconomic disadvantage to attained years of schooling.

### 3.7 Providing context: Description of schooling aspirations and reasons for not meeting them

CLHNS participants had high aspirations for school attainment. When interviewed when the children were 11 years old, 98 % of mothers who were high school graduates, and 58 % of mothers with 6 or fewer years of schooling wanted their child to attain a college degree or more, and aspirations were not different for sons versus daughters. Children of mothers who aspired for them to be college graduates or higher completed, on average, 2.4 years more education (1.08 years when adjusted for the full set of early life SED variables noted above). As adolescents and young adults, about 84 % of females and 68 % of males aspired to finish college or more. At age 18–19 years, 27 % of those who expressed a schooling aspiration felt they could not attain it, with nearly half saying it was because of financial reasons. Among those with stated aspirations and valid final schooling (N = 1900), 26.1 % of females and 22.2 % of males met their aspiration to attain a college degree or higher.

In 1994 and subsequent surveys, participants (mothers in 1994, the adolescent/young adult in subsequent waves) who not enrolled were asked why they were not in school. For the 107 children not in school in 1994, mothers most often said it was because the child had lost interest in school (39 %), was ill or injured (19.6 %) or there were financial constraints (12.1 %). In 1998, among the 457 adolescents not enrolled in school, lost interest (40.9 %) remained the most frequent reason, followed by financial reasons (32.4 %). By 2002, when participants were ages 18–19 years, the most frequent reason for not being enrolled (n = 1207) was financial constraints (55.6 %) followed by lost interest (21.6 %), preferring to work (8.0 %), already having children (4%) and embarrassment (3.6 %). The latter relates to being much older than classmates, for example, an 18-year old who would have to go back to the first year of high school where classmates would typically be 13–14 years old. Across all years, household income and assets were significantly lower among those citing financial constraints compared to the other frequent category of “lost interest”.

Among mothers or caregivers of elementary age school children, 40.4 % said someone in the household read to the child (of these 60 % read one or more times per week, 40 % only occasionally) and 57.4 % said they had children’s books at home. Nearly all children had school homework, but nearly 30 % received no help with homework from any family members. Among those who received help, the mother was the most frequent helper, followed by older siblings. However, provision of help for homework and presence of books in the home were unrelated to school attainment.

## 4 Discussion

This paper offers a comprehensive view of how school attainment relates to early life and continued disadvantage along the schooling life course. Final attained schooling is the result of a series of decisions, from on-time school entry through completion of higher education. Thus, it is important to explore those decisions using detailed prospective longitudinal data. The CLHNS, our long-running community-based study in Cebu, Philippines, provides rich data from multiple survey rounds from the prenatal period through age 35 years when participants have completed schooling.

### 4.1 LAZ and rIQ interrelationships and their association with schooling

Consistent with many other studies, we use early child linear growth represented by LAZ at about age 2 years as a marker of chronic early life disadvantage, and child IQ as an indicator of early child cognitive development. LAZ and IQ have separate as well as shared underlying determinants of poverty, morbidity, undernutrition and suboptimal care and stimulation (Grantham-McGregor et al., 2007) (Prado et al., 2019a). In our multivariable analyses, we treat these well-known factors as mutual confounders and estimate the separate and combined associations of LAZ and rIQ with school attainment.

We first estimated linear regression models of total years of attained schooling and found that early life LAZ and IQ had nearly identical beta-coefficients which were unchanged when mutually adjusted and their interaction was not significant. This suggests independent roles for each of these important early life risk factors consistent with work done by Prado et al. (Prado et al., 2019a). Our estimate that each unit difference in early life LAZ is associated with 0.4 years of final schooling is similar to estimates from other COHORTS collaboration studies in LMICs (Victora et al., 2008), but larger than the net effect observed for age 1 year LAZ in the Young Lives Study (Fink and Rockers, 2014). Using Demographic and Health Survey data combined to create a synthetic panel from 425 birth cohorts across 21 LMICs, researchers (Karra and Fink, 2019) reported an average 0.28 year deficit per unit of HAZ, but the wide 95 % CI of this estimate (− 0.68–1.22) led to their judgement that the evidence was inconclusive. Differences in the estimated associations of early life LAZ with later outcomes may reflect, in part, the degree to which underlying determinants of LAZ are measured and taken into account.

In the CLHNS, child IQ was measured at a mean age of age 8.5 years, when most children were already in school. The IQ test used in the CLHNS is sensitive to time in school, and we therefore used a residual which is independent of schooling in our regression models and a path from years of schooling to IQ in our SEM. rIQ was significantly associated with total years of schooling, and increased likelihood of school continuation after elementary school. In our path model, there was a significant path from LAZ to IQ, and thus LAZ has direct and indirect associations (through IQ) with final years of schooling. The model cannot reveal whether this association reflects unmeasured confounding, or a possible causal connection. It may also be the case that parents and teachers have different expectations for taller children who may be perceived as more mature.

The independent associations of LAZ and rIQ in our regression models and our path model suggest that strategies to enhance child cognitive development can have important consequences for schooling even without improving linear growth (Frongillo et al., 2019; Leroy and Frongillo, 2019; Leroy et al., 2013; Leroy et al., 2013). However, nutrition interventions to improve linear growth may not necessarily result in improved cognition unless accompanied by child development components (Prado et al., 2019a).

### 4.2 Improved growth after age two

In a prior study using the CLHNS data, we found that change in LAZ at age 2 to HAZ at age 8 was related to higher IQ (Mendez and Adair, 1999). In the current analysis, change is related to more total school years but the sequential logit results suggest that this is attributable to associations with completion of elementary school.

Other work in South Africa found that children with recovery from stunting between ages 2 and 5 years did not have better cognitive performance compared to those who remained stunted (Casale and Desmond, 2016), suggesting that improved linear growth alone does not necessarily translate to improved cognitive development.

### 4.3 Preschool

Consistent with work in Peru (Cueto et al., 2016) and other settings (Behrman et al., 2014), we found that children who attended preschool or kindergarten had higher IQ scores and higher attained schooling, irrespective of their LAZ.: Prior to the 1990 deployment of government-funded day care centers in each community for children aged 6 and below (RA 6972, 2009) preschool education was an expense shouldered by households and may have entailed other costs such as transportation, depending on the location of the school. However, we adjusted for SES factors in our models, and thus our finding of a significant association of preschool with higher school attainment supports the Philippine government’s early child development program and initiatives to have all children participate in preschool programs, including legislation (RA 10157, 2012) which institutionalized kindergarten into the Basic Education System and further strengthened by legislation that established the Enhanced Basic Education system (K to 12) making kindergarten education compulsory (RA 10533, 2013).

### 4.4 Contributions from the continuation models

A key contribution of this paper is the continuation model approach, which has been used by economists and sociologists to understand how family background and SES affect schooling (Saweda and Lokshin, 1999; Mare, 2011). The key premise is that final schooling status represents cumulative decisions about staying in or returning to school over time. Unlike linear regression models of final attained schooling that capture the entire history of past influences at once, using the continuation approach, we estimate how early life factors relate to moving on to each major school level separately. Studies using this approach tend to find weakening associations of early life family circumstances or child characteristics across schooling transitions (Mare, 2011). These findings may be biased by selection. The eligible sample for each sequential model declines owing to drop out, which is related to the early life characteristics of interest. The magnitude of bias has led to a recommendation to limit the use of such models to description and avoid causal inference that might inform policy (Mare, 2011). However, a World Bank study in Pakistan which found waning gender effects across continuation decisions found that estimates were not markedly changed when complex methods to account for selection bias were used (Saweda and Lokshin, 1999).

While early life LAZ was significantly associated with total years of schooling, it was not consistently associated with all schooling transitions. The strongest associations (highest ORs) were for completing elementary school and the transition to college after high school. Thus, once students have completed high school, early life stunting is of less importance. However, it is important to note that shorter children are less and less represented in each sequential continuation model, which may affect the size of estimates. In contrast, rIQ remains significantly associated with all school transitions after completing elementary school.

This emphasizes the importance of making sure that disadvantaged children complete elementary education, consistent with SDG 4 (Grantham-McGregor and Cumper, 1992).

A weakness of the Seqlogit continuation model is that it does not accommodate time-varying predictors. Thus, we extended the approach to include sequential estimates of continuation models using time varying family characteristics. The models demonstrate persistence of early life LAZ and IQ associations, even in the face of changing household circumstances. Household wealth and parental education were consistent predictors of continuation at each level. Child hospitalizations, presence of older siblings and household income were not associated with continuation. Addition of younger siblings reduced the likelihood that elementary school graduates would go on to high school, perhaps explained by the notion of competition for household resources (Saweda and Lokshin, 1999). Moving to a different community only influenced the likelihood of completing elementary school. Upon completing high school, marriage markedly reduced the likelihood of going on to college. In 2002, at age 18.5, 15 % of females and 5% of males were married. Marriage and childbearing may be viewed as constraints to further education, given competing time and economic demands. Of note is the lack of interaction of time-varying variables with early life LAZ and rIQ: enablers and constraints had similar consequences regardless of early life status. Across the sequential logit models, parental education remained a strong predictor of school continuation and final school attainment. Such strong associations speak to the potential for intergenerational benefits as the more highly educated members of the CLHNS cohort are likely to influence the education of their offspring. (Behrman et al., 2017).

### 4.5 Descriptive analysis of qualitative data

The detailed questionnaires administered to caregivers (mostly mothers) when children were 11 years old, and to adolescents and young adults provide important qualitative information about schooling. Caregiver’s educational aspirations for their children are very high, even among those with low education. Adolescent and young adult aspirations were similar, but unfortunately, aspirations for most exceeded their attainment. Among adolescents, loss of interest in school was the top reason given for early drop out and for not reaching the ultimate level of education to which they aspired. Among young adults, financial barriers to going on to college were most often cited as reasons for not continuing schooling. Findings from our study are consistent with the Philippine Government’s 2013 Functional Literacy, Education, and Mass Media Survey report which cited that among all participants ages 6–24 years, the top reason for not being in school was “looking for work”, followed by financial reasons and lack of interest (National Statistics Office, 2013). We continue to observe the same trend in a nationally representative sample of Filipino children age 10 years in 2016: a higher proportion of 10-year old males reported not aspiring for a college education compared to females (21.9 % vs 14.6 %) (Alegado et al., 2020).

The notion of “lost interest” speaks to the importance of school curricula which keep students engaged in learning.

### 4.6 Limitations and strengths

Our study has several important limitations. First, as with any long-running cohort study, attrition and missing data may introduce selection bias. To maximize sample size, we included data from participants who were not enrolled in school at their last completed visit. This may have resulted in misclassification of some participants who went back to school after leaving Metro Cebu. Migration from the Metro Cebu area was the main reason for loss to follow-up between 1991 and 2018, and migrants were more likely to have been from families with higher education, wealth, and from more urban communities. However, use of IPW to account for attrition did not produce markedly different results, suggesting that despite these differences, the relationships of interest were not different those lost to follow-up vs retained in the analysis sample.

Despite the detailed information collected in the CLHNS, we are likely to be missing some key factors associated with early life LAZ, IQ and schooling, resulting in residual confounding. For example, the significant path from early life LAZ to IQ in our SEM may represent unmeasured underlying determinants such as other aspects of health, or factors related to motivation or other types of social and emotional skills.

We used a measure of fluid abilities assessed by a non-verbal intelligence test administered at a mean age of 8.5 years. This presents two main challenges. First, other important aspects of child cognitive development and abilities relevant for school attainment are not fully captured with this measure. Second, IQ was measured after many children had been in school for 1 or more years, and the measure was strongly related to time in school. We used an IQ residual, independent of years in elementary school, but an ideal measure would be one administered prior to any school enrolment.

We focus on the postnatal period. However, exploratory analyses of the role of size and gestational age at birth showed that these birth outcomes were not significant predictors of school attainment once we account for LAZ and rIQ (both of which are related to birth size).

Key strengths of the study include characteristics of the survey and data, such as the detailed repeated measures across participant’s lives, the relatively large sample size, and the use of final schooling status. In addition, we used analytic methods which allowed comparison with other studies, but also provided insights into the varying impacts of early life factors across the full range of schooling decisions made from school entry to completion of higher education degrees.

This work adds support to the promotion of optimal nutrition, health, and development in early childhood as means to enhance school attainment, a key aspect of human capital. High school completion rates in the Philippines in 2005, when our participants were 21 years of age were 61.7 % (UNESCO, 2015) but have now improved to 78.2 % (World Bank, 2018). Attention not only to early life circumstances but to continued economic well-being of families and to school quality will be needed to achieve the SDG4 target of secondary education for all.

## Author statements

**Linda Adair**: Conceptualization, Methodology, Formal Analysis, Writing-Original Draft, Review and Editing, Funding Acquisition. **Judith Borja**: Conceptualization, Investigation, Writing-Review and Editing, Funding Acquisition**. Delia Carba**: Conceptualization, Investigation, Writing-Review and Editing**. Nanette Lee**: Conceptualization, Writing-Review and Editing, Funding Acquisition.

## Funding

The Cebu Longitudinal Health and Nutrition Survey has received funding from multiple sources over its 35-year history, including the 10.13039/100000002National Institutes of Health (most recently TW05596; HL085144; and HD054501), the Nestle Coordinating Center for Nutrition Research, Wyeth International, The 10.13039/100000010Ford Foundation, The US National Academy of Science, The 10.13039/100004423World Health Organization, The US Agency for International Development (via grants from Wellstart International, the International Center for Research on Women, Family Health International, MEASURE), The 10.13039/100004425Asian Development Bank, The 10.13039/100004421World Bank, The 10.13039/100005627Thrasher Research Fund, The Mellon Foundation, Nestle Research Foundation, The 10.13039/100000001National Science Foundation, The Wenner Gren Foundation, and The Carolina Population Center and its NIH Center grant (P2C HD050924). The most recent CLHNS follow-up survey was funded by the 10.13039/100000865Bill and Melinda Gates FoundationOPP1164115.

## Role of the funding sources

Grants provided resources for data collection and management, and support for investigator time. Funders had no involvement in the study design in the analysis and interpretation of data; in the writing of the report; and in the decision to submit the article for publication.

## Declaration of Competing Interest

The authors report no declarations of interest.
