# Supplementary material for: Stunting, IQ, and final school attainment in the Cebu Longitudinal Health and Nutrition Survey birth cohort
Source: Econ Hum Biol. 2021 Aug;42:100999. doi: 10.1016/j.ehb.2021.100999 (PMC8222184; doi:10.1016/j.ehb.2021.100999)
Supplement: Supplementary file 1 [file mmc1.docx]

| Supplemental Table 1. Likelihood of advancing to each subsequent education level associated with early life child LAZ, IQ, and household, community and family circumstances | | | | | | | | | | |
| --- | --- | --- | --- | --- | --- | --- | --- | --- | --- | --- |
|  | Completed Elementary | | Some High School | | High School Graduate | | Some College | | College Graduate | |
|  | OR | 95% CI | OR | 95% CI | OR | 95% CI | OR | 95% CI | OR | 95% CI |
| LAZ | 1.35^***^ | 1.14,1.60 | 1.15 | 0.94,1.40 | 1.21^**^ | 1.05,1.38 | 1.30^***^ | 1.15,1.48 | 1.17 | 0.98,1.40 |
| rIQ | 1.17 | 0.97,1.41 | 1.38^**^ | 1.12,1.71 | 1.19^*^ | 1.03,1.37 | 1.33^***^ | 1.17,1.51 | 1.20^*^ | 1.01,1.41 |
| Male | 0.32^***^ | 0.22,0.47 | 0.46^***^ | 0.31,0.69 | 0.47^***^ | 0.36,0.62 | 0.87 | 0.68,1.11 | 0.58^***^ | 0.41,0.80 |
| Firstborn | 1.25 | 0.74,2.13 | 0.95 | 0.55,1.64 | 0.98 | 0.69,1.38 | 1.05 | 0.78,1.42 | 0.69 | 0.47,1.01 |
| Urbanicity Index | 1.05 | 0.91,1.21 | 1.03 | 0.87,1.21 | 0.90 | 0.81,1.01 | 1.09 | 0.98,1.21 | 0.90 | 0.78,1.05 |
| HH Assets | 1.13 | 0.96,1.33 | 1.18 | 0.99,1.40 | 1.09 | 0.98,1.20 | 1.19^***^ | 1.10,1.30 | 1.08 | 0.98,1.19 |
| HH Income Q1 (ref) |  |  |  |  |  |  |  |  |  |  |
| HH Income Q2 | 1.10 | 0.71,1.69 | 1.48 | 0.84,2.61 | 1.34 | 0.88,2.02 | 1.04 | 0.70,1.55 | 0.62 | 0.33,1.16 |
| HH Income Q3 | 1.85^*^ | 1.09,3.13 | 1.31 | 0.75,2.30 | 1.28 | 0.84,1.95 | 1.00 | 0.67,1.49 | 1.32 | 0.72,2.41 |
| HH Income Q4 | 1.81^*^ | 1.01,3.27 | 2.02^*^ | 1.04,3.92 | 1.16 | 0.74,1.80 | 1.44 | 0.95,2.20 | 1.49 | 0.81,2.72 |
| HH Income Q5 | 1.48 | 0.71,3.07 | 2.08 | 0.89,4.88 | 0.93 | 0.56,1.54 | 1.39 | 0.85,2.26 | 1.49 | 0.77,2.87 |
| HH Size (# persons) | 1.03 | 0.95,1.11 | 0.95 | 0.88,1.03 | 0.96 | 0.91,1.01 | 0.94^*^ | 0.89,0.99 | 0.96 | 0.90,1.02 |
| Mother's height | 1.00 | 0.96,1.03 | 0.98 | 0.94,1.03 | 1.02 | 0.99,1.05 | 0.98 | 0.96,1.01 | 1.03 | 0.99,1.06 |
| Mother's Education <6 years (ref) |  |  |  |  |  |  |  |  |  |  |
| Elementary Only (6 years) | 1.57^*^ | 1.04,2.37 | 1.33 | 0.82,2.18 | 1.22 | 0.86,1.73 | 1.14 | 0.81,1.60 | 0.90 | 0.52,1.56 |
| Some High School | 2.94^***^ | 1.66,5.24 | 1.67 | 0.94,2.98 | 1.19 | 0.82,1.73 | 1.49^*^ | 1.05,2.11 | 0.80 | 0.47,1.39 |
| High School Graduate | 4.82^*^ | 1.07,21.75 | 2.35 | 0.65,8.54 | 3.56^**^ | 1.66,7.62 | 3.66^***^ | 2.18,6.14 | 0.89 | 0.48,1.68 |
| Dad Education Missing | 0.81 | 0.34,1.94 | 0.94 | 0.35,2.52 | 1.17 | 0.58,2.37 | 1.48 | 0.78,2.83 | 1.55 | 0.66,3.64 |
| <6 yrs (ref) |  |  |  |  |  |  |  |  |  |  |
| Elementary Only (6 years) | 1.43 | 0.93,2.22 | 0.98 | 0.60,1.59 | 0.90 | 0.63,1.29 | 1.43 | 1.00,2.06 | 1.36 | 0.75,2.47 |
| Some High School | 2.20^**^ | 1.30,3.71 | 2.06^*^ | 1.12,3.78 | 1.16 | 0.79,1.69 | 1.75^**^ | 1.23,2.48 | 1.55 | 0.88,2.74 |
| High School Graduate | 3.81^*^ | 1.10,13.21 | 3.72^*^ | 1.05,13.26 | 2.17^*^ | 1.16,4.07 | 2.06^**^ | 1.27,3.33 | 2.48^**^ | 1.29,4.76 |
| Readers in the home | 1.44 | 1.00,2.09 | 0.88 | 0.58,1.35 | 1.11 | 0.83,1.50 | 1.03 | 0.78,1.35 | 1.11 | 0.75,1.64 |

| \| Supplemental Table 2. Likelihood of advancing to each subsequent education level associated with early life child LAZ, IQ, and contemporaneously measured household, community and family circumstances \| \| \| \| \| \| \| \| \| \| \| \| --- \| --- \| --- \| --- \| --- \| --- \| --- \| --- \| --- \| --- \| --- \| \|  \| Completed Elementary \| \| Some High School \| \| High School Graduate \| \| Some College \| \| College Graduate \| \| \|  \| OR \| 95% CI \| OR \| 95% CI \| OR \| 95% CI \| OR \| 95% CI \| OR \| 95% CI \| \|  \|  \|  \|  \|  \|  \|  \|  \|  \|  \|  \| \| LAZ \| 1.23^*^ \| 1.03,1.48 \| 1.07 \| 0.87,1.31 \| 1.20^**^ \| 1.04,1.38 \| 1.36^***^ \| 1.19,1.56 \| 1.16 \| 0.97,1.38 \| \| rIQ \| 1.11 \| 0.92,1.35 \| 1.33^**^ \| 1.08,1.65 \| 1.15 \| 0.99,1.33 \| 1.28^***^ \| 1.11,1.46 \| 1.21^*^ \| 1.01,1.44 \| \| Male \| 0.33^***^ \| 0.23,0.49 \| 0.47^***^ \| 0.31,0.71 \| 0.42^***^ \| 0.32,0.56 \| 0.83 \| 0.64,1.07 \| 0.58^**^ \| 0.41,0.80 \| \| Firstborn \| 1.79 \| 0.97,3.29 \| 1.12 \| 0.59,2.11 \| 1.14 \| 0.77,1.68 \| 1.34 \| 0.93,1.92 \| 0.85 \| 0.54,1.34 \| \| Mother's height \| 1 \| 0.96,1.04 \| 0.98 \| 0.94,1.03 \| 1.02 \| 0.99,1.05 \| 0.98 \| 0.95,1.01 \| 1.02 \| 0.99,1.06 \| \| Mother's Education <6 years (ref) \|  \|  \|  \|  \|  \|  \|  \|  \|  \|  \| \| Elementary Only (6 years) \| 1.57^*^ \| 1.03,2.40 \| 1.23 \| 0.75,2.02 \| 1.24 \| 0.86,1.77 \| 1.05 \| 0.74,1.50 \| 0.87 \| 0.49,1.53 \| \| Some High School \| 2.87^***^ \| 1.59,5.17 \| 1.43 \| 0.80,2.56 \| 1.16 \| 0.80,1.67 \| 1.39 \| 0.97,1.99 \| 0.78 \| 0.45,1.34 \| \| High School Graduate \| 3.78 \| 0.85,16.89 \| 2.04 \| 0.56,7.43 \| 2.96^**^ \| 1.39,6.31 \| 3.71^***^ \| 2.17,6.34 \| 0.95 \| 0.51,1.78 \| \| Dad Education Missing \| 0.95 \| 0.37,2.41 \| 1.09 \| 0.40,2.98 \| . \|  \| 1.81 \| 0.89,3.69 \| 1.89 \| 0.77,4.63 \| \| <6 yrs (ref) \|  \|  \|  \|  \|  \|  \|  \|  \|  \|  \| \| Elementary Only (6 years) \| 1.49 \| 0.94,2.35 \| 1 \| 0.60,1.64 \| 0.65 \| 0.31,1.36 \| 1.33 \| 0.91,1.95 \| 1.38 \| 0.75,2.54 \| \| Some High School \| 1.93^*^ \| 1.13,3.31 \| 2.05^*^ \| 1.10,3.83 \| 0.76 \| 0.37,1.59 \| 1.67^**^ \| 1.16,2.40 \| 1.69 \| 0.95,3.00 \| \| High School Graduate \| 3.18 \| 0.91,11.11 \| 3.79^*^ \| 1.05,13.67 \| 1.39 \| 0.58,3.33 \| 1.83^*^ \| 1.11,3.02 \| 2.75^**^ \| 1.42,5.34 \| \| Urbanicity Index \| 1 \| 0.99,1.01 \| 0.99 \| 0.98,1.01 \| 0.99 \| 0.98,1.00 \| 1.02^**^ \| 1.01,1.03 \| 0.98^*^ \| 0.97,1.00 \| \| HH Income Q1 (ref) \|  \|  \|  \|  \|  \|  \|  \|  \|  \|  \| \| HH Income Q2 \| 0.76 \| 0.48,1.20 \| 0.95 \| 0.56,1.63 \| 1.04 \| 0.69,1.59 \| 0.98 \| 0.63,1.53 \| 1.12 \| 0.57,2.18 \| \| HH Income Q3 \| 0.78 \| 0.47,1.31 \| 1.89 \| 0.95,3.76 \| 1.05 \| 0.68,1.63 \| 1.05 \| 0.67,1.64 \| 1.33 \| 0.68,2.58 \| \| HH Income Q4 \| 1.31 \| 0.68,2.52 \| 0.91 \| 0.47,1.74 \| 1.01 \| 0.63,1.62 \| 1.08 \| 0.69,1.70 \| 0.9 \| 0.47,1.71 \| \| HH Income Q5 \| 0.9 \| 0.40,2.03 \| 1.16 \| 0.49,2.77 \| 0.72 \| 0.42,1.25 \| 1.47 \| 0.90,2.41 \| 1.4 \| 0.72,2.70 \| \| HH Assets \| 1.52^***^ \| 1.32,1.75 \| 1.33^***^ \| 1.15,1.54 \| 1.25^***^ \| 1.15,1.37 \| 1.36^***^ \| 1.25,1.48 \| 1.21^***^ \| 1.09,1.35 \| \| Younger siblings \| 0.92 \| 0.81,1.04 \| 0.82^**^ \| 0.70,0.94 \| 0.97 \| 0.90,1.06 \| 0.96 \| 0.89,1.04 \| 0.93 \| 0.83,1.03 \| \| Older siblings \| 1.01 \| 0.88,1.17 \| 0.9 \| 0.77,1.05 \| 1.06 \| 0.93,1.21 \| 1.03 \| 0.90,1.18 \| 1.06 \| 0.88,1.27 \| \| Hospitalized \| 0.81 \| 0.52,1.27 \| 1.26 \| 0.71,2.23 \| 0.76 \| 0.43,1.34 \| 1.32 \| 0.70,2.49 \| 0.69 \| 0.36,1.34 \| \| Mom Absent \| 0.94 \| 0.40,2.24 \| 0.84 \| 0.34,2.09 \| 2.02^*^ \| 1.14,3.59 \| 1.39 \| 0.87,2.20 \| 1.69 \| 0.93,3.08 \| \| Dad Absent \| 1.08 \| 0.57,2.05 \| 0.58 \| 0.31,1.09 \| 1.17 \| 0.77,1.78 \| 1.4 \| 0.97,2.01 \| 1.23 \| 0.79,1.93 \| \| Moved residence \| 0.50^*^ \| 0.26,0.98 \| 0.7 \| 0.33,1.46 \| 1.43 \| 0.86,2.40 \| 0.88 \| 0.51,1.49 \| 0.9 \| 0.44,1.83 \| \| Got married \|  \|  \|  \|  \|  \|  \| 0.40^**^ \| 0.21,0.78 \| 0.63 \| 0.21,1.86 \| |
| --- | --- | --- | --- | --- | --- | --- | --- | --- | --- | --- | --- | --- | --- | --- | --- | --- | --- | --- | --- | --- | --- | --- | --- | --- | --- | --- | --- | --- | --- | --- | --- | --- | --- | --- | --- | --- | --- | --- | --- | --- | --- | --- | --- | --- | --- | --- | --- | --- | --- | --- | --- | --- | --- | --- | --- | --- | --- | --- | --- | --- | --- | --- | --- | --- | --- | --- | --- | --- | --- | --- | --- | --- | --- | --- | --- | --- | --- | --- | --- | --- | --- | --- | --- | --- | --- | --- | --- | --- | --- | --- | --- | --- | --- | --- | --- | --- | --- | --- | --- | --- | --- | --- | --- | --- | --- | --- | --- | --- | --- | --- | --- | --- | --- | --- | --- | --- | --- | --- | --- | --- | --- | --- | --- | --- | --- | --- | --- | --- | --- | --- | --- | --- | --- | --- | --- | --- | --- | --- | --- | --- | --- | --- | --- | --- | --- | --- | --- | --- | --- | --- | --- | --- | --- | --- | --- | --- | --- | --- | --- | --- | --- | --- | --- | --- | --- | --- | --- | --- | --- | --- | --- | --- | --- | --- | --- | --- | --- | --- | --- | --- | --- | --- | --- | --- | --- | --- | --- | --- | --- | --- | --- | --- | --- | --- | --- | --- | --- | --- | --- | --- | --- | --- | --- | --- | --- | --- | --- | --- | --- | --- | --- | --- | --- | --- | --- | --- | --- | --- | --- | --- | --- | --- | --- | --- | --- | --- | --- | --- | --- | --- | --- | --- | --- | --- | --- | --- | --- | --- | --- | --- | --- | --- | --- | --- | --- | --- | --- | --- | --- | --- | --- | --- | --- | --- | --- | --- | --- | --- | --- | --- | --- | --- | --- | --- | --- | --- | --- | --- | --- | --- | --- | --- | --- | --- | --- | --- | --- | --- | --- | --- | --- | --- | --- | --- | --- | --- | --- | --- | --- | --- | --- | --- | --- | --- | --- | --- | --- | --- | --- | --- | --- | --- | --- | --- | --- | --- | --- | --- | --- | --- | --- | --- | --- | --- | --- | --- | --- | --- | --- | --- | --- | --- | --- | --- | --- | --- | --- | --- | --- | --- | --- | --- | --- | --- | --- | --- | --- | --- | --- | --- | --- | --- | --- | --- | --- | --- | --- | --- | --- | --- | --- | --- |
